# Hybrid Sparse Transformer and Wavelet Fusion-Based Deep Unfolding Network for Hyperspectral Snapshot Compressive Imaging

**DOI:** 10.3390/s24196184

**Published:** 2024-09-24

**Authors:** Yangke Ying, Jin Wang, Yunhui Shi, Nam Ling

**Affiliations:** 1 Beijing Key Laboratory of Multimedia and Intelligent Software Technology, Beijing Institute of Artificial Intelligence, School of Information Science and Technology, Beijing University of Technology, Beijing 100124, China; yingyangke@emails.bjut.edu.cn; 2 Beijing Institute of Artificial Intelligence, School of Computer Science, Beijing University of Technology, Beijing 100124, China; ijinwang@bjut.edu.cn; 3 Department of Computer Science and Engineering, Santa Clara University, Santa Clara, CA 95053, USA; nling@scu.edu

**Keywords:** compressive sensing, hyperspectral image reconstruction, snapshot compressive imaging, deep unfolding network

## Abstract

Recently, deep unfolding network methods have significantly progressed in hyperspectral snapshot compressive imaging. Many approaches directly employ Transformer models to boost the feature representation capabilities of algorithms. However, they often fall short of leveraging the full potential of self-attention mechanisms. Additionally, current methods lack adequate consideration of both intra-stage and inter-stage feature fusion, which hampers their overall performance. To tackle these challenges, we introduce a novel approach that hybridizes the sparse Transformer and wavelet fusion-based deep unfolding network for hyperspectral image (HSI) reconstruction. Our method includes the development of a spatial sparse Transformer and a spectral sparse Transformer, designed to capture spatial and spectral attention of HSI data, respectively, thus enhancing the Transformer’s feature representation capabilities. Furthermore, we incorporate wavelet-based methods for both intra-stage and inter-stage feature fusion, which significantly boosts the algorithm’s reconstruction performance. Extensive experiments across various datasets confirm the superiority of our proposed approach.

## 1. Introduction

The continuous demand for capturing high-dimensional data has driven the development of imaging devices and processing algorithms. Compared to imaging systems that acquire RGB images, hyperspectral imaging (HSI) systems can capture a wider range of wavelength information from scenes. As a result, they have rapidly developed and been applied to various downstream visual tasks [1,2,3,4,5,6]. Traditional HSI systems primarily acquire scene information through one-dimensional or two-dimensional scanning mechanisms [7]. However, these systems have slow imaging speeds due to the need for multiple exposures, making them unsuitable for dynamic scenes. In recent years, many snapshot compressive imaging (SCI) systems [8,9,10] have emerged with the advancement of compressive sensing theory [11]. These systems can obtain three-dimensional HSI data from two-dimensional observations with a single exposure. Among SCI systems, coded aperture snapshot spectral imaging (CASSI) systems [9] have gained widespread use due to their advantages of low cost, low power consumption, and high sampling rates. Specifically, CASSI systems consist of two main components: a hardware encoder and a software decoder. The hardware encoder uses optical devices like coded apertures and prisms to modulate three-dimensional HSI scenes into two-dimensional compressed measurements. The primary task of the software decoder is to reconstruct the original three-dimensional HSI scenes from the acquired two-dimensional measurements. The quality of data reconstruction entirely depends on the effectiveness of the algorithm used. Therefore, the core challenge of this research is to reconstruct higher-quality original HSI data.

Current reconstruction algorithms can be broadly categorized into two types: traditional model-based optimization methods and learning-based deep neural network methods. Model-based methods typically use various handcrafted priors to solve traditional ill-posed optimization inverse problems [12,13,14,15,16,17,18,19,20]. While these methods benefit from mathematical derivations, enhancing their theoretical interpretability, they face limitations in prior design and often exhibit slow reconstruction speeds. On the other hand, deep learning techniques, with their powerful feature modeling capabilities, have shown outstanding performance in many tasks. Consequently, many learning-based algorithms have been applied to HSI reconstruction to overcome the limitations of model-based methods. Learning-based methods can be further divided into three strategies: end-to-end (E2E) methods, plug-and-play (PnP) methods, and deep unfolding network (DUN) methods. E2E methods [21,22,23,24,25,26,27,28]. use deep networks to directly establish a mapping between three-dimensional HSI data and two-dimensional compressed measurements. Although E2E methods have achieved some impressive reconstruction results, they still suffer from a lack of interpretability due to the black-box nature of the networks. PnP methods [29,30,31,32,33] integrate deep network modules into model-based methods, replacing traditional handcrafted priors with deep network modules. While PnP methods more effectively address the prior subproblem in optimization models, they still follow the traditional optimization process and do not fundamentally overcome the limitations of model-based methods. DUN methods [34,35,36,37,38,39,40,41,42,43,44,45,46,47,48,49,50] construct multi-stage unfolding networks to implement the iterative solving process of model-based methods in an end-to-end manner. DUN methods alleviate the interpretability challenges and provide encouraging experimental results. As a result, DUN methods are rapidly evolving and showing great potential.

Despite the promising reconstruction results, existing DUN methods still face several challenges in HSI reconstruction. On the one hand, many existing DUN methods utilize the non-local information modeling capabilities of Transformer [51] modules to significantly enhance the final reconstruction performance. Existing Transformers typically model the correlations among all tokens in the query–key pairs. However, in practice, if some tokens in the query do not correlate with tokens in the key, the estimated self-attention values for these tokens are still used for feature aggregation, thereby limiting the final feature representation capability. Additionally, sparse self-attention mechanisms have demonstrated outstanding performance in numerous RGB image processing tasks [52,53]. On the other hand, DUN methods experience feature information loss within each network stage due to cross-scale transformations, as well as across different stages due to frequent signal-to-feature conversions. In existing methods, Herosnet [42] mitigates cross-stage information loss by concatenating features from earlier stages and passing them into the next stage. PADUT [45] achieves cross-stage information fusion by applying Fourier transform operations to the features from a frequency-domain perspective. RDLUF [46] reduces intra-stage multi-scale information loss through the convolutional fusion of features at different scales, while also minimizing cross-stage information loss by generating modulation parameters from previous stage features to guide information capture in the next stage. SUCTNet [54] introduces a dual transformer-based module to simultaneously utilize HSI interactions and details at both global and local scales. EDUNet! [49] enhances cross-stage feature transfer efficiency by constructing a cross-stage spectral self-attention network module that leverages HSI characteristics. M2U-Net [55] achieves feature fusion for the HSI super-resolution task through a cross-attention guided module. uHNTC [56] designs a multi-level cross-feature attention mechanism to achieve hierarchical spatial–spectral feature fusion for the hyperspectral and multispectral image fusion task. Recently, wavelet-based feature fusion methods have been applied to many low-level vision tasks [57,58] and have achieved impressive results. This prompts us to explore how to leverage the properties of the wavelet transform [59] to construct a feature fusion module for HSI data, aimed at addressing the issue of intra-stage and cross-stage feature information loss.

To address these challenges, we propose a novel method that hybridizes the sparse Transformer and wavelet fusion-based deep unfolding network for hyperspectral snapshot compressive imaging. Specifically, we introduce a sparse spatial Transformer and a sparse spectral Transformer to model the self-attention in the spatial and spectral dimensions, respectively. By extracting the most relevant regions through sparse operations to compute similarity for feature aggregation, we enhance the feature representation capability of the Transformer. Additionally, we utilize the cross-scale properties of the wavelet transform to construct a wavelet-based intra-stage feature fusion module, addressing the intra-stage feature information loss in DUN methods. Finally, we further leverage the wavelet transform to build an inter-stage feature fusion module, enabling cross-stage feature transmission in the wavelet domain and avoiding the information loss caused by frequent signal-to-feature conversions. The main contributions of this study can be summarized as follows:

- We propose a novel method that hybridizes the sparse Transformer and wavelet fusion-based deep unfolding network for hyperspectral snapshot compressive imaging.
- To enhance the expressive capability of existing Transformer methods, we propose a sparse spatial–spectral Transformer. This approach uses sparse operations to avoid calculating correlations for irrelevant tokens during feature aggregation.
- To address the issue of information loss within and across stages of the DUN method, we design the wavelet-based intra-stage fusion module and wavelet-based inter-stage fusion module, respectively. These fusion modules utilize the characteristics of the wavelet transform to enhance HSI reconstruction.

## 2. Related Works

### 2.1. Model-Based Traditional Optimization Methods

For reconstruction methods in the hyperspectral image snapshot compressive imaging task, traditional model-based optimization approaches incorporate various hand-crafted prior knowledge as regularization terms and solve the ill-posed inverse problem through iterative optimization. For instance, GAP-TV [12] incorporates the total variation (TV) prior term and utilizes the generalized alternating projection (GAP) [60] algorithm to address the optimization problem for HSI reconstruction. To exploit the sparse constraint of HSI for reconstruction, Lin et al. [13] suggests learning an overcomplete dictionary that represents HSI more sparsely than previous methods. The paper [14] leverages the spatial and spectral sparsity properties of HSI data to develop a dictionary learning approach based on sparse priors for HSI scene reconstruction. The paper [15] compares the impact of various estimation algorithms on the effectiveness of HSI reconstruction. CT3D [16] introduces a method for reconstructing HSI using coupled tensor decomposition and the alternating direction method of multipliers (ADMM) [61] iteration. MMLE-GMM [17] extends a maximum marginal likelihood estimator to a Gaussian mixture model with a primarily low-rank covariance matrix, achieving accelerated optimization for reconstruction. DeSCI [18] creates a joint model that integrates non-local self-similarity and rank minimization methods with the SCI sensing process, resulting in excellent reconstruction outcomes. NGmeet [20] proposes an algorithm that combines the global spectral low-rank property and spatial non-local self-similarity prior for hyperspectral image reconstruction. Traditional model-based iterative optimization methods, relying on limited prior designs and requiring multiple iterations for optimization, lead to slow reconstruction speeds and suboptimal outcomes.

### 2.2. Learning-Based Neural Network Methods

Due to the powerful non-linear feature modeling capabilities of deep neural networks, many learning-based methods are being employed to address the reconstruction of HSI data. These learning-based methods can be categorized into three strategies: (1) end-to-end (E2E) methods; (2) plug-and-play (PnP) methods; and (3) deep unfolding network (DUN) methods.

(1) The E2E methods directly model the mapping relationship between 2D compressed measurements and 3D HSI data through end-to-end learning using deep networks. For instance, λ-net [23] sets up a two-stage network, where the initial reconstruction stage utilizes self-attention for reconstruction, followed by a refinement stage to further improve the results. TSA-Net [24] tackles different dimensions separately through the use of spatial–spectral self-attention mechanisms. MST [25] introduces an innovative Mask-guided Spectral-wise Transformer designed for reconstructing HSI. HDNet [26] integrates dual-domain constraints in frequency and spatial–spectral domains within its objective function to improve the quality of reconstruction outcomes. CST [27] introduces a novel coarse-to-fine sparse Transformer approach that incorporates the sparsity of hyperspectral imaging into deep learning for reconstruction purposes. BIRNAT [28] integrates the expressive capabilities of an end-to-end convolutional framework with bidirectional recurrent neural networks (RNNs) to effectively capture sequence correlations in snapshot compressive imaging. However, the end-to-end (E2E) methods encounter challenges in interpretability because of the opaque nature of convolutional networks.

(2) The PnP methods often use the outcomes from pre-trained denoising networks to replace solving the prior subproblem in traditional model-based approaches. PnP-HSI [29] employs a deep learning denoising network as a regularization prior and addresses the reconstruction optimization problem using the ADMM algorithm. PnP-DIP-HSI [30] integrates a deep image prior (DIP) network as a prior within the iterative optimization algorithm, establishing a self-supervised framework for reconstructing HSI. Qiu et al. [31] extends the PnP algorithm by incorporating deep image denoising and total variation priors into the conventional optimization objective. LR2DP [32] and LRSDN [33] leverage the robust spectral correlation and complex spatial structures inherent in HSI for SCI reconstruction, integrating model-driven low-rank priors with data-driven deep priors. The PnP methods provide a partial solution to the interpretability limitations of E2E methods, yet they continue to grapple with the inherent challenge of slow reconstruction speeds characteristic of traditional model-based approaches.

(3) The DUN methods usually utilize multi-stage networks instead of the iterative optimization processes seen in model-based methods. Each stage employs deep neural network models to address both the data and prior subproblems iteratively. For instance, GAP-Net [34] transforms the generalized alternating projection optimization algorithm into a multi-stage network tailored for HSI reconstruction. ADMM-Net [35] unfolds the standard tensor alternating direction method of multipliers optimization algorithm into a multi-layer network structure. DSSP [36] enhances the spatial–spectral fidelity of HSI data by leveraging the local coherence and dynamic features of HSI to construct prior learning. DNU [37] creates a regularization term by exploring both local and non-local correlations of HSI as data-driven priors. Zhang et al. [38] proposes developing a deep Canonical Polyadic decomposition model in the unfolded network to learn a low-rank prior for HSI data. DGSMP [39] combines a learnable Gaussian Scale Mixture prior with the Maximum A Posteriori estimation algorithm to achieve HSI reconstruction. DGSM-Swin [40], a variant of DGSM [39], is constructed by leveraging the Swin Transformer [62], further enhancing the reconstruction performance of HSI. GAP-CCot [41] combines the expressive capabilities of convolution and the content Transformer, creating a hybrid network module that is integrated into the GAP algorithm for SCI reconstruction. HerosNet [42] unfolds the Iterative Shrinkage-Thresholding Algorithm (ISTA) [63] into a multi-stage network, inserting learnable flexible sensing matrices and constructing adaptive dynamic gradient descent at each stage. Ying et al. [43] employ a dual-domain feature learning approach to comprehensively acquire complementary information in the feature space, enhancing the overall algorithm’s feature modeling capability. DAUHST [44] introduces an unfolding network framework that is aware of degradation, where parameters estimated during the degradation process govern different iterative stages. PADUT [45] designs a framework incorporating pixel-adaptive steps and a non-local spectral Transformer to enhance expressive capability at each stage. RDLUF [46] introduces a strategy for residual degradation learning that links the sensing matrix with the degradation process. This integration enhances spectral–spatial representation capabilities by incorporating both spectral and spatial priors. D^2^PL-Net [47] endeavors to dynamically learn the actual degradation matrix throughout the deep unfolding network process to enhance HSI reconstruction, thereby narrowing the disparity between ideal and real-world degradations. DADF-Net [48] integrates the underlying connection between the network input and the true HSI, introducing a dynamic Fourier network to achieve high-quality HSI reconstruction. EDUNet [49] innovatively introduces a memory-assisted descent method based on momentum acceleration and a cross-stage spectral self-attention network to model the gradient-driven update module and the proximal mapping module, respectively. DPU [50] implements an HSI reconstruction model based on dual prior unfolding, which improves iteration efficiency by jointly utilizing multiple deep priors while strategically incorporating focused attention into the framework to enhance reconstruction quality. The DUN methods combine interpretability with high-quality reconstruction. Nonetheless, the current methods can still be enhanced by improving feature expression and addressing the challenge of cross-stage information loss.

## 3. Method

### 3.1. Preliminary

**Figure 1:**
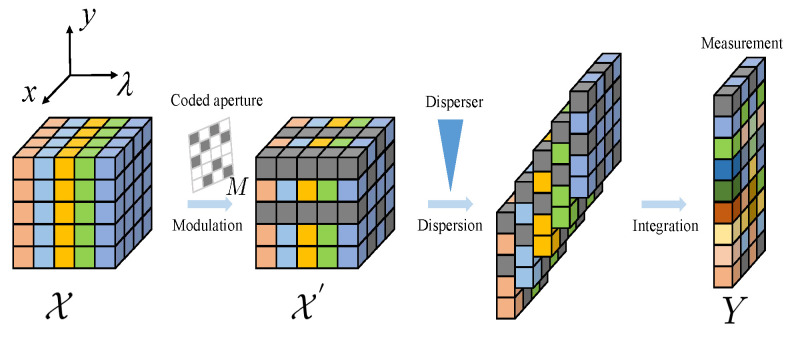
The physical imaging process of the SD-CASSI system for HSI data.

The complete physical imaging process for the Single-Disperser Coded Aperture Snapshot Spectral Imaging (SD-CASSI) system for hyperspectral image (HSI) data is shown in Figure 1. The SD-CASSI system is composed of a set of physical optical devices with different functions, designed to compress 3D HSI data into 2D compressed measurements. In detail, as the objective lens acquires 3D hyperspectral data, each spectral band undergoes modulation along the spatial dimension by the coded aperture. The modulated data subsequently pass through an optical dispersion element to shift each spectral band. Ultimately, all the spectral bands after dispersion are superimposed along the spectral direction to obtain the processed 2D compression measurement data. The 3D HSI data are denoted as $X\in R^{H\times W\times N_{\lambda }}$, where *H*, *W* and $N_{\lambda }$ represent the height, width and spectral bands of 3D HSI data, respectively. The matrix $M\in R^{H\times W}$ stands for the coded aperture. Hence, the physical imaging process of the 3D HSI can be described as follows:

(1) $$\begin{array}{c} X^{\prime }\left(:,:,n_{\lambda }\right)=X\left(:,:,n_{\lambda }\right)⊙M \end{array}$$

where $X^{\prime }\in R^{H\times W\times N_{\lambda }}$ denotes the modulated cube, $n_{\lambda }\in \left[1,\ldots ,N_{\lambda }\right]$ indexes the spectral bands, and ⊙ denotes the element-wise product. The modulated HSI data, after being spatially shifted and summed element-wise across different spectral bands, can be represented as follows:

(2) $$\begin{array}{c} Y\left(m,n\right)=\sum_{n_{\lambda }=1}^{N_{\lambda }}X^{\prime }\left(m,n+d_{\lambda },n_{\lambda }\right)+N \end{array}$$

where $Y\in R^{H\times (W+N_{\lambda }-1)}$ represents the 2D compressive measurement; *m* and *n* represent the spatial coordinates; and $d_{\lambda }$ represents the shifting distance. $N\in R^{H\times (W+N_{\lambda }-1)}$ represents the noise. In summary, the vectorized representation of the SD-CASSI system for 3D HSI data is described as follows:

(3) $$\begin{array}{c} y=\Phi x+n \end{array}$$

where $x\in R^{HWN_{\lambda }}$, $y\in R^{H(W+N_{\lambda }-1)}$, $n\in R^{H(W+N_{\lambda }-1)}$ denote the vectorized form of X, *Y*, and *N*, respectively. $\Phi \in R^{H\left(W+N_{\lambda }-1\right)\times HWN_{\lambda }}$ denotes the sensing matrix. After modeling the system’s imaging model, the primary challenge of the HSI snapshot compressive imaging task is to reconstruct the original 3D HSI scene x given y and Φ. In the following sections, we will separately introduce the overall framework of the algorithm, the sparse spatial–spectral Transformer module, the wavelet-based intra-stage fusion module, and the wavelet-based inter-stage fusion module.

### 3.2. Overall Algorithm Framework

**Figure 2:**
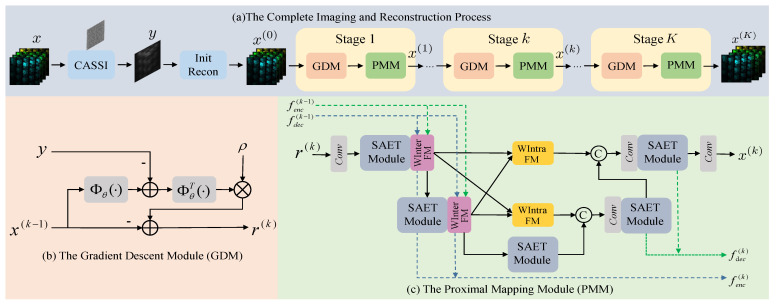
Overall algorithm framework. (**a**) The complete imaging and reconstruction process of the system. (**b**) The network structure of the gradient descent module. (**c**) The network structure of the proximal mapping module.

The overall imaging and reconstruction process is shown in Figure 2a. For the given raw HSI datum x, the observed datum y is obtained after passing through the CASSI imaging system. Subsequently, the overall reconstruction process can be modeled as the following optimization problem:

(4) $$\begin{array}{c} x=\underset{x}{\arg\min}\frac{1}{2}{∥y-\Phi x∥}_{2}^{2}+\lambda \psi \left(x\right) \end{array}$$

where $\frac{1}{2}{∥y-\Phi x∥}_{2}^{2}$ represents the data fidelity term in the optimization problem, ψ(x) represents the prior term, and λ represents the weighting parameter. Subsequently, the proximal gradient descent algorithm is employed to solve this ill-posed optimization inverse problem. Specifically, the overall optimization problem is transformed into a gradient descent operation and a proximal mapping operation. The solution process is as follows:

(5) $$\begin{array}{cc} \;\;\;\;\;r^{\left(k\right)} & =x^{\left(k-1\right)}-\rho \Phi^{⊤}\left(\Phi x^{\left(k-1\right)}-y\right) \end{array}$$

(6) $$\begin{array}{cc} x^{\left(k\right)} & ={\mathrm{prox}}_{\lambda ,\psi }\left(r^{\left(k\right)}\right) \end{array}$$

where Equation (5) represents the gradient descent operation, and Equation (6) represents the proximal mapping operation. Here, $x^{\left(k-1\right)}$ and $x^{\left(k\right)}$ denote the reconstruction results at iteration k−1 and k, respectively. The variable ρ represents the step size, and $r^{\left(k\right)}$ represents the intermediate variable.

Through the iterative optimization process of $x^{\left(k\right)}$ and $r^{\left(k\right)}$, the final *x* can be reconstructed. Subsequently, we unfold the different iterative processes into different stages of deep unfolding network architecture, introducing gradient descent modules and proximal mapping modules to replace traditional operations. As shown in Figure 2a, each stage of the unfolded network contains a gradient descent module and a proximal mapping module. The structure of the gradient descent module is shown in Figure 2b. To enhance the representative capacity of the overall network framework, we construct $\Phi_{\theta }\left(\cdot \right)$ and $\Phi_{\theta }^{⊤}\left(\cdot \right)$ by adding residual networks after the operations of matrices Φ and $\Phi^{⊤}$. The structure of the proximal mapping module is shown in Figure 2c. We adopt a conventional U-Net structure, constructing a sparse spatial–spectral Transformer (SAET) module in the encoder–decoder parts of each layer to enhance the feature representation capabilities. Additionally, we introduce the Wavelet-based Intra-stage Fusion Module (WIntraFM) and Wavelet-based Inter-stage Fusion Module (WInterFM) to reduce feature loss within and across stages. The detailed structures of the different modules are described in the following sections.

### 3.3. The Sparse Spatial–Spectral Transformer (SAET) Module

**Figure 3:**
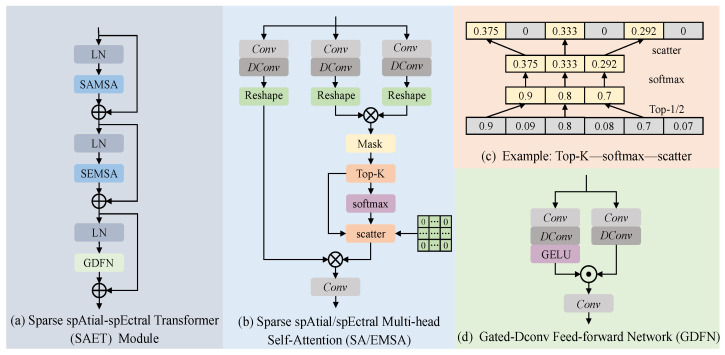
(**a**) The structure of the sparse spatial–spectral Transformer module. (**b**) The structure of the sparse spatial/spectral multi-head self-attention. (**c**) A specific example of a correlation map sparsification. (**d**) The detailed structure of the gated-dconv feed-forward network.

The overview of the sparse spatial–spectral Transformer module is shown in Figure 3a. LN represents the Layer Normalization layer, SAMSA and SEMSA represent Sparse Spatial Multi-Head Self-Attention and Sparse Spectral Multi-Head Self-Attention, respectively, and GDFN represents the Gated-Dconv Feed-forward Network. We compose the final SAET module by serially connecting different network parts and utilizing skip connections.

The structures of SAMSA and SEMSA are shown in Figure 3b. Both SAMSA and SEMSA use the same method to transform the input features into queries, keys, and values. The difference lies in the way they compute the self-attention maps. Specifically, for a given input feature $X_{in}\in R^{H\times W\times C}$, SA/EMSA employs a convolution layer Conv with 1×1 kernels and a depth-wise convolution layer DConv with 3×3 kernels to embed $X_{in}$ and generate $Q\in R^{H\times W\times C}$, $K\in R^{H\times W\times C}$, and $V\in R^{H\times W\times C}$. The formulas are expressed as follows:

(7) $$\begin{array}{c} Q=W_{d}^{Q}W_{p}^{Q}X_{in},K=W_{d}^{K}W_{p}^{K}X_{in},V=W_{d}^{V}W_{p}^{V}X_{in} \end{array}$$

where $W_{p}^{\left(\cdot \right)}$ and $W_{q}^{\left(\cdot \right)}$ represent the weight parameters of the Conv layer and the DConv layer, respectively.

For SAMSA, the input features are split along the spatial dimension into non-overlapping windows of size M×M. Each pixel within a window is treated as a token, and self-attention maps are computed within each window. Therefore, the query *Q*, key *K*, and value *V* features can be reshaped into $Q_{A},K_{A},V_{A}\in R^{\frac{HW}{M^{2}}\times M^{2}\times C}$. Subsequently, the $Q_{A}$, $K_{A}$, and $V_{A}$ features are split along the last dimension into *h* heads and represented as: $Q_{A}=\left[Q_{A}^{1},\cdots ,Q_{A}^{h}\right]$, $K_{A}=\left[K_{A}^{1},\cdots ,K_{A}^{h}\right]$, $V_{A}=\left[V_{A}^{1},\cdots ,V_{A}^{h}\right]$. The dimension of each head is $d\frac{h}{A}=\frac{C}{h}$. The query $Q_{A}^{i}$ and key $K_{A}^{i}$ are dot-multiplied to obtain a self-attention map Mask of size $\frac{HW}{M^{2}}\times M^{2}\times M^{2}$, representing the correlation of all pixels within each window. However, in practice, not all pixels are correlated. By applying sparsification to extract only the relevant pixels for correlation computation, the expressiveness of the self-attention mechanism can be further enhanced. Specifically, for the original self-attention map Mask, we use a ‘Top-k’ operation to select the top-*k* pixels with the highest correlation. Then, we apply ‘Softmax’ normalization to compute correlation coefficients for the selected pixels. Finally, we use a ‘scatter’ operation to return the correlation coefficients to the corresponding positions in the original self-attention map Mask, replacing positions where no correlation coefficient exists with zeros. SAMSA can be formally expressed as follows:

(8) $$\begin{array}{cc} Mask & =Q_{A}^{i}{\left(K_{A}^{i}\right)}^{⊤}\;\;\;\;\;\; \end{array}$$

(9) $$\begin{array}{cc} {\mathrm{head}}_{i} & =\mathrm{SparseAtten}(Q_{A}^{i},K_{A}^{i},V_{A}^{i}) \end{array}$$

(10) $$\begin{array}{cc} & =\mathrm{scatter}\left(\mathrm{Softmax}\left(\frac{\text{Top-k}(\mathrm{Mask})}{\sqrt{d_{A}^{h}}}\right)\right)V_{A}^{i} \end{array}$$

(11) $$\begin{array}{cc} \;\;\;\;X_{out} & =W_{p}\mathrm{Concat}\left({\mathrm{head}}_{1},\cdots ,{\mathrm{head}}_{n}\right)+X_{\mathrm{in}} \end{array}$$

where ‘SparseAtten’ represents the sparse self-attention operation, ‘Concat’ represents the feature concatenation operation, $W_{p}$ represents the convolutional layer operation, and $X_{out}\in R^{H\times W\times C}$ represents the feature output. To better understand the sparsification operation, we present an example of the ‘Top-k’ to ‘Softmax’ to ‘scatter’ operations as shown in Figure 3c.

For SEMSA, the spatial dimensions of the input features are transformed into column vectors of size HW. Each feature channel is treated as a token, and the self-attention map is obtained by calculating the correlations among all channels. Therefore, the query *Q*, key *K*, and value *V* features can be reshaped into $Q_{E},K_{E},V_{E}\in R^{HW\times C}$. Subsequently, we split $Q_{E}$, $K_{E}$ and $V_{E}$ features along the spectral channel dimension into *h* heads, represented as $Q_{E}=\left[Q_{E}^{1},\cdots ,Q_{E}^{h}\right]$, $K_{E}=\left[K_{E}^{1},\cdots ,K_{E}^{h}\right]$, $V_{E}=\left[V_{E}^{1},\cdots ,V_{E}^{h}\right]$. The dimension of each head is $d\frac{h}{E}=\frac{C}{h}$. Next, the self-attention map Mask of size $\frac{C}{h}\times \frac{C}{h}$ for each head can be obtained by the dot product of $Q_{E}^{i}$ and $K_{E}^{i}$, representing the correlations among all channels in the spectral dimension. After obtaining the self-attention map Mask, we apply the same sparsification operation as in SAMSA to extract the correlated channels and compute the correlation coefficients. Finally, the formal expression of the SEMSA block is as follows:

(12) $$\begin{array}{cc} Mask & ={\left(K_{E}^{i}\right)}^{⊤}Q_{E}^{i}\;\;\;\;\;\; \end{array}$$

(13) $$\begin{array}{cc} {\mathrm{head}}_{i} & =\mathrm{SparseAtten}(Q_{E}^{i},K_{E}^{i},V_{E}^{i}) \end{array}$$

(14) $$\begin{array}{cc} & =\mathrm{scatter}\left(\mathrm{Softmax}\left(\frac{\text{Top-k}(\mathrm{Mask})}{\sqrt{d_{E}^{h}}}\right)\right)V_{E}^{i} \end{array}$$

(15) $$\begin{array}{cc} \;\;\;\;\;X_{out} & =W_{p}\mathrm{Concat}\left({\mathrm{head}}_{1},\cdots ,{\mathrm{head}}_{n}\right)+X_{\mathrm{in}} \end{array}$$

### 3.4. The Wavelet-Based Intra-Stage Fusion Module (WIntraFM)

**Figure 4:**
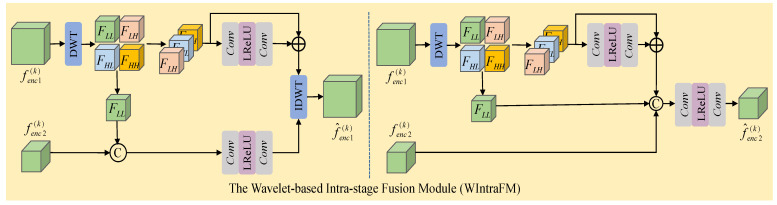
The wavelet-based intra-stage fusion module.

Since the proximal mapping network adopts a U-Net structure, there are up-sampling and down-sampling operations in different encoder–decoder features, leading to feature information loss. Discrete wavelet transform inherently possesses scale down-sampling characteristics, so we construct a wavelet-based intra-stage feature fusion module. The specific structure is shown in Figure 4.

For two encoder output features $f_{enc1}^{\left(k\right)}$ and $f_{enc2}^{\left(k\right)}$ at different spatial scales, we apply the wavelet transform operation only to the large-scale feature $f_{enc1}^{\left(k\right)}$ to leverage the multi-scale characteristics of the wavelet transform. The expression is as follows:

(16) $$\begin{array}{c} F_{enc1}^{LL},F_{enc1}^{LH},F_{enc1}^{HL},F_{enc1}^{HH},=\mathrm{DWT}\left(f_{\mathrm{enc}1}^{\left(k\right)}\right) \end{array}$$

where ‘DWT’ represents the discrete wavelet transform. In this paper, we specifically use the Haar wavelet transform. $F_{enc1}^{LL}$ denotes the low-frequency component of the feature $f_{enc1}^{\left(k\right)}$, while $\left[F_{enc1}^{LH},F_{enc1}^{HL},F_{enc1}^{HH}\right]$ represents the high-frequency components of the feature $f_{enc1}^{\left(k\right)}$. Due to the reduced scale of the features after the wavelet transform, the spatial resolution of the feature $f_{enc2}^{\left(k\right)}$ matches that of the features $\left[F_{enc1}^{LL},F_{enc1}^{LH},F_{enc1}^{HL},F_{enc1}^{HH}\right]$. Subsequently, we perform feature fusion on features of the same size to reduce information loss.

To obtain the high-resolution encoder feature after fusion, we concatenate the low-frequency component $F_{enc1}^{LL}$ with feature $f_{enc2}^{\left(k\right)}$ and perform a low-frequency enhancement operation to obtain the fused low-frequency information. Additionally, we enhance the high-frequency components $\left[F_{enc1}^{LH},F_{enc1}^{HL},F_{enc1}^{HH}\right]$ separately to obtain the fused high-frequency information. Finally, we perform an inverse wavelet transform on the fused low-frequency and high-frequency features to obtain the final fused feature. The formal expression is as follows:

(17) $${\hat{F}}_{L}={\mathrm{Conv}}_{3\times 3}(\mathrm{LRelu}\left({\mathrm{Conv}}_{1\times 1}\left(\mathrm{Concat}\left(f_{enc2}^{\left(k\right)},F_{enc1}^{\mathrm{LL}}\right)\right)\right))$$

(18) $${\hat{F}}_{H}=\left[F_{enc1}^{LH},F_{enc1}^{HL},F_{enc1}^{HH}\right]+{\mathrm{Conv}}_{1\times 1}\left(\mathrm{LRelu}\left({\mathrm{Conv}}_{1\times 1}\left(\left[F_{enc1}^{\mathrm{LH}},F_{enc1}^{\mathrm{HL}},F_{enc1}^{\mathrm{HH}}\right]\right)\right)\right)$$

(19) $$\begin{array}{cc} \; & {\hat{f}}_{enc1}^{\left(k\right)}=\mathrm{IDWT}\left({\hat{F}}_{L},{\hat{F}}_{H}\right) \end{array}$$

where ‘${\mathrm{Conv}}_{1\times 1}$’ and ‘${\mathrm{Conv}}_{3\times 3}$’ represent convolutional layers with kernel sizes of 1 × 1 and 3 × 3, respectively. ‘Concat’ represents the feature concatenation operation, ‘LRelu’ represents the activation function, and ‘IDWT’ represents the inverse discrete wavelet transform. The variables ${\hat{F}}_{L}$ and ${\hat{F}}_{H}$ represent the fused low-frequency and high-frequency features, respectively, and ${\hat{f}}_{enc1}^{\left(k\right)}$ represents the output high-resolution fused feature.

To obtain the low-resolution encoder feature after fusion, we first enhance the high-frequency components $\left[F_{enc1}^{LH},F_{enc1}^{HL},F_{enc1}^{HH}\right]$ separately to get the fused high-frequency component ${\hat{F}}_{H}^{\prime }$. Next, we concatenate the fused ${\hat{F}}_{H}^{\prime }$ with the low-frequency component $F_{enc1}^{LL}$ and feature $f_{enc2}^{\left(k\right)}$, and further enhance these features to obtain the final low-resolution fused feature ${\hat{f}}_{enc2}^{\left(k\right)}$. The formal expression is as follows:

(20) $$\begin{array}{cc} {\hat{F}}_{H}^{\prime }= & \left[F_{enc1}^{LH},F_{enc1}^{HL},F_{enc1}^{HH}\right]+{\mathrm{Conv}}_{1\times 1}\left(\mathrm{LRelu}\left({\mathrm{Conv}}_{1\times 1}\left(\left[F_{enc1}^{\mathrm{LH}},F_{enc1}^{\mathrm{HL}},F_{enc1}^{\mathrm{HH}}\right]\right)\right)\right) \end{array}$$

(21) $$\begin{array}{cc} \; & {\hat{f}}_{enc2}^{\left(k\right)}={\mathrm{Conv}}_{3\times 3}\left(\mathrm{LRelu}\left({\mathrm{Conv}}_{1\times 1}\left(\mathrm{Concat}\left({\hat{F}}_{H}^{\prime },F_{enc1}^{\mathrm{LL}},f_{enc2}^{\left(k\right)}\right)\right)\right)\right) \end{array}$$

### 3.5. The Wavelet-Based Inter-Stage Fusion Module (WInterFM)

**Figure 5:**
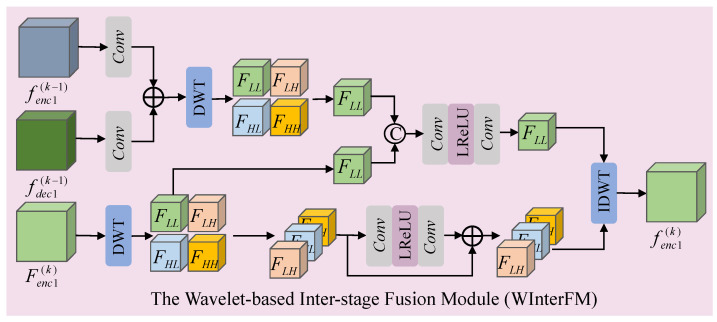
The wavelet-based inter-stage fusion module.

In the deep unfolding network, the continuous transformation of signals to features across different stages leads to feature information loss. Wavelet transform can effectively learn and emphasize high-frequency details in features. Therefore, we design a wavelet-based inter-stage feature fusion module to mitigate the information loss across stages. The specific structure is shown in Figure 5.

Specifically, taking the inter-stage fusion operation of the high-resolution encoder feature $F_{enc1}^{\left(k\right)}$ at the *k* stage as an example, we first add the encoder feature $f_{enc1}^{\left(k-1\right)}$ and decoder feature $f_{dec1}^{\left(k-1\right)}$ from the k−1 stage to obtain feature $F^{\left(k-1\right)}$. Then, we perform a discrete wavelet transform on feature $F^{\left(k-1\right)}$ to obtain the high-frequency components $\left[F_{LH}^{\left(k-1\right)},F_{HL}^{\left(k-1\right)},F_{HH}^{\left(k-1\right)}\right]$ and low-frequency component $F_{LL}^{\left(k-1\right)}$. Additionally, we perform a wavelet transform on the feature $F_{enc1}^{\left(k\right)}$ from the *k* stage to obtain the high-frequency components $\left[F_{LH}^{\left(k\right)},F_{HL}^{\left(k\right)},F_{HH}^{\left(k\right)}\right]$ and low-frequency component $F_{LL}^{\left(k\right)}$. Next, we concatenate the low-frequency components $F_{LL}^{\left(k\right)}$ and $F_{LL}^{\left(k-1\right)}$, followed by a low-frequency enhancement to obtain the low-frequency component $f_{LL}^{\left(k\right)}$ of the inter-stage fusion feature. To reduce noise impact, we only perform high-frequency feature enhancement on the high-frequency components $\left[F_{LH}^{\left(k\right)},F_{HL}^{\left(k\right)},F_{HH}^{\left(k\right)}\right]$ from stage *k* to obtain the fused high-frequency components $\left[f_{LH}^{\left(k\right)},f_{HL}^{\left(k\right)},f_{HH}^{\left(k\right)}\right]$. Finally, we perform an inverse wavelet transform on the high-frequency components $\left[f_{LH}^{\left(k\right)},f_{HL}^{\left(k\right)},f_{HH}^{\left(k\right)}\right]$ and low-frequency component $f_{LL}^{\left(k\right)}$ to obtain the final inter-stage fusion feature $f_{enc1}^{\left(k\right)}$. The formal expressions are as follows:

(22) $$F^{\left(k-1\right)}={\mathrm{Conv}}_{3\times 3}(f_{enc1}^{\left(k-1\right)})+{\mathrm{Conv}}_{3\times 3}(f_{dec1}^{\left(k-1\right)})$$

(23) $$F_{LL}^{\left(k-1\right)},F_{LH}^{\left(k-1\right)},F_{HL}^{\left(k-1\right)},F_{HH}^{\left(k-1\right)}=\mathrm{DWT}(F^{\left(k-1\right)})$$

(24) $$\begin{array}{cc} \; & F_{LL}^{\left(k\right)},F_{LH}^{\left(k\right)},F_{HL}^{\left(k\right)},F_{HH}^{\left(k\right)}=\mathrm{DWT}(F_{enc1}^{\left(k\right)}) \end{array}$$

(25) $$f_{LL}^{\left(k\right)}={Conv}_{3\times 3}(LRelu({Conv}_{1\times 1}(Concat\left(F_{LL}^{\left(k\right)},F_{LL}^{\left(k-1\right)}\right))))$$

(26) $$[f_{LH}^{\left(k\right)},f_{HL}^{\left(k\right)},f_{HH}^{\left(k\right)}]={Conv}_{1\times 1}\left(LRelu\left({Conv}_{1\times 1}\left(\left[F_{LH}^{\left(k\right)},F_{HL}^{\left(k\right)},F_{HH}^{\left(k\right)}\right]\right)\right)\right)+\left[F_{LH}^{\left(k\right)},F_{HL}^{\left(k\right)},F_{HH}^{\left(k\right)}\right]$$

(27) $$\begin{array}{cc} \; & f_{enc1}^{\left(k\right)}=IDWT\left(f_{LL}^{\left(k\right)},\left[f_{LH}^{\left(k\right)},f_{HL}^{\left(k\right)},f_{HH}^{\left(k\right)}\right]\right) \end{array}$$

where ‘${\mathrm{Conv}}_{1\times 1}$’ and ‘${\mathrm{Conv}}_{3\times 3}$’ represent convolutional layers with kernel sizes of 1 × 1 and 3 × 3, respectively. ‘Concat’ represents the feature concatenation operation, ‘LRelu’ represents the activation function, and ‘DWT’ and ‘IDWT’ represent the discrete wavelet transform and inverse transform.

## 4. Experimental Results

In this section, we conduct experiments on various datasets to validate the superiority of our method compared to other state-of-the-art methods. The specific content includes experimental setup, simulation results, real data results, and ablation study, among other subsections.

### 4.1. Experiment Setup

Simulated experiments on CAVE and KAIST datasets: The CAVE dataset [64] contains 32 HSI samples, each with spatial dimensions of 512 × 512. The KAIST dataset [65] consists of 30 HSI samples, each with spatial dimensions of 2704 × 3376. Both datasets feature 31 spectral bands, spanning wavelengths from 400 nm to 700 nm with 10 nm intervals. To ensure consistency with the experimental setups of other state-of-the-art methods, we also employ spectral interpolation to adjust each HSI sample to 28 spectral bands within the wavelength range of 450 nm to 650 nm. In the actual training and testing process, we maintain the same experimental settings as other state-of-the-art methods. The CAVE dataset is randomly cropped into HSI patches consistent with the size of the coded aperture to construct the training set. The coded aperture size is 256 × 256, and all training HSI patches are of size 256 × 256 × 28. Meanwhile, we select HSI patches of size 256 × 256 × 28 from 10 scenes in the KAIST dataset as the test set. At this point, the spatial receptive field of our proposed network is 256 × 256.

Simulated experiments on ARAD_1K dataset: The ARAD_1K dataset [66] provides a large-scale natural hyperspectral image dataset. Each HSI sample in the ARAD_1K dataset consists of 31 spectral bands, with a spatial resolution of 482 × 512, and covers a wavelength range of 400–700 nm. It contains 1000 hyperspectral images, of which 900 are used as the training set and 50 as the test set. The size of the coded aperture is set to 256 × 256, and each HSI block in the ARAD_1K training set is cropped to a size of 256 × 256 × 31. Similar to the KAIST dataset, our ARAD_1K test dataset is also constructed by cropping HSI patches of size 256 × 256 × 31 from 10 scenes.

Real-world scene dataset: For the real-world dataset, we use the 2D measurements from five real-scene samples provided by TSA-Net [24] to assess the effectiveness of our proposed method. Each 2D measurement has dimensions of 660 × 714, and the coded aperture is sized at 660 × 660. To build the training dataset, we randomly crop the CAVE and KAIST datasets into data blocks of size 660 × 660 × 28.

Experimental settings: We use Root Mean Square Error (RMSE) as the loss function for the algorithm. Additionally, all related experiments are constructed based on the PyTorch deep learning framework and conducted on an NVIDIA RTX 3090 GPU. The algorithm is trained using the Adam optimizer with an initial learning rate set to 0.0001 and a maximum of 200 training epochs. The spectral shift step for all HSI data is configured to 2. We also use the Peak Signal-to-Noise Ratio (PSNR) and Structural Similarity (SSIM) Index [67] as metrics to evaluate the reconstruction performance of different algorithms. PSNR and SSIM serve as two standards for spatial quality assessment, measuring visual quality and structural similarity, respectively. Higher values for both PSNR and SSIM indicate improved spatial reconstruction.

### 4.2. Simulation Results on CAVE and KAIST

The objective results comparison for the KAIST dataset test scenes is shown in Table 1. We select nine state-of-the-art methods as comparison methods, including four E2E methods (TSA-Net [24], HDNet [26], MST [25], and CST [27]) and five DUN methods (DGSMP [39], HerosNet [42], DAUHST [44], PADUT [45], and RDLUF [46]). We present the PSNR and SSIM results for all comparison methods, with the best and second-best reconstruction results for all test scenes highlighted in red and blue, respectively. In addition, we calculate the number of parameters (Params) and floating-point operations (FLOPs) to evaluate the model complexity of all comparison methods. As seen in Table 1, our method achieves the best average reconstruction results across all test scenes and delivers the best reconstruction results in most of the individual test scenes. Meanwhile, our method also shows a certain advantage in complexity compared to other methods. Our method achieves a PSNR of 39.76 dB and an SSIM of 0.979. Specifically, compared to the current leading E2E method, CST [27], our results demonstrate an improvement of 3.91 dB in PSNR and 0.0025 in SSIM. Furthermore, compared to the top DUN method, RDLUF [46], our approach improves PSNR by 0.31 dB and SSIM by 0.002. Therefore, these objective results validate the effectiveness of our method on the KAIST test dataset.

**Figure 6:**
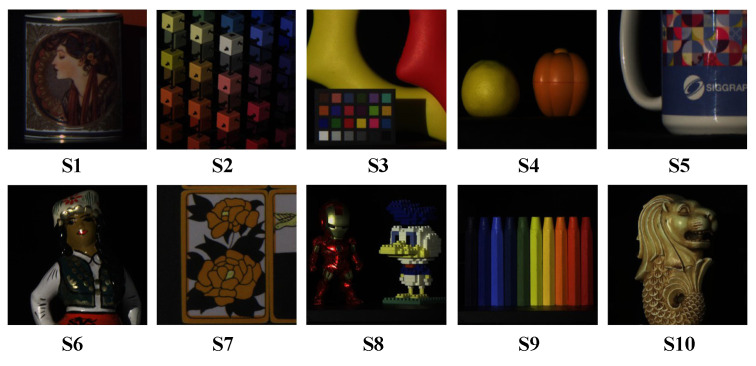
RGB images of ten test scenes from the KAIST dataset.

**Figure 7:**
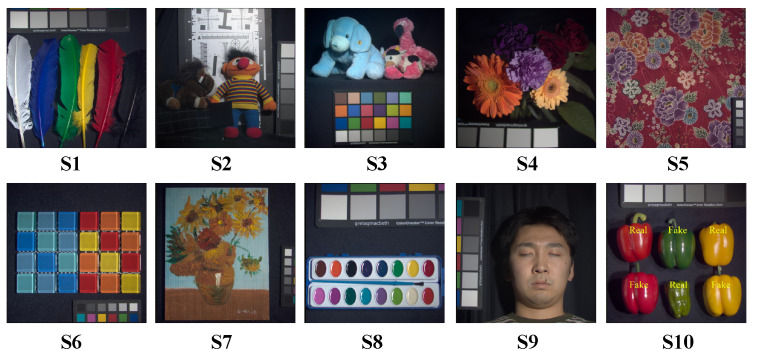
RGB images of 10 training scenes from the CAVE dataset.

**Figure 8:**
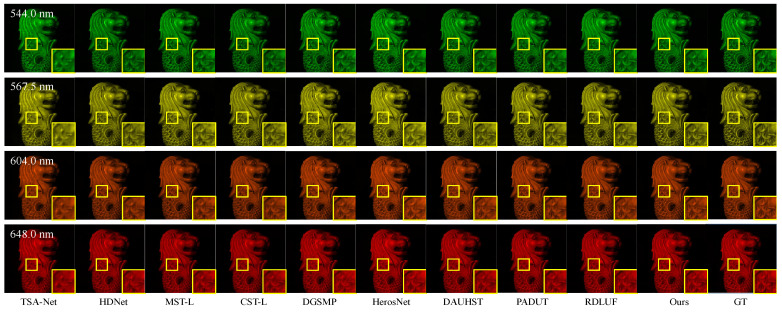
Comparison of the reconstruction results for scene S10 in the KAIST test dataset using different methods. The enlarged regions help to compare the reconstruction results better.

To illustrate the qualitative results on the KAIST test dataset, we present a subjective comparison of the reconstruction outcomes for the test scenes using different methods. To better illustrate the comparison between the training and test datasets, we present the RGB images of 10 test scenes from the KAIST dataset and 10 training scenes from the CAVE dataset, shown in Figure 6 and Figure 7, respectively. The subjective comparison of the reconstruction results for scene S10 in the KAIST dataset is shown in Figure 8. We select data from four different spectral bands for the reconstruction results and compare the subjective effects of all methods. We crop and enlarge the yellow box areas in each spectral band image to facilitate a clearer comparison of the reconstruction differences. From Figure 8, it is evident that our proposed method exhibits clearer texture details across different spectral bands compared to other methods. This further validates the effectiveness of our approach on the KAIST test dataset.

**Figure 9:**
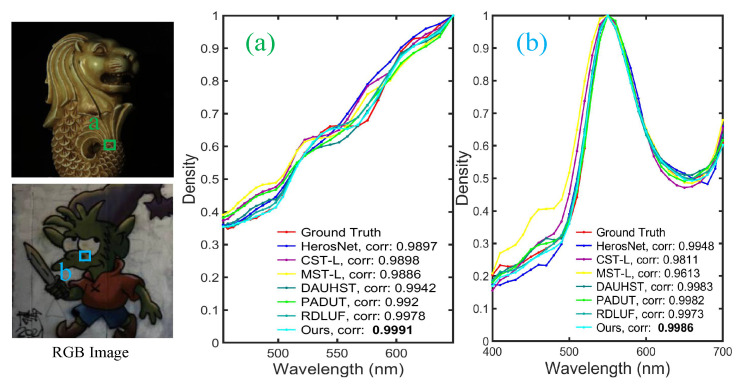
Spectral density curves of reconstruction results for KAIST test scene S10 and ARAD_1K test scene S5 using different methods. (**a**,**b**) The spectral density curves and correlation coefficients, respectively, for the selected regions.

Additionally, to further compare the spectral consistency of reconstruction results from different methods, we select a small region from test scene S10 for evaluation as shown in Figure 9a. We present the spectral density curves of different reconstruction results and calculate the correlation coefficients between the reconstructed results and the ground truth data. Our method’s reconstruction results are the closest to the ground truth spectral density curve and achieve the highest correlation coefficient, further validating the accuracy of our reconstruction.

### 4.3. Simulation Results on ARAD_1K

The objective results comparison for the ARAD_1K dataset test scenes is shown in Table 2. We select nine state-of-the-art methods as comparison methods, including four E2E methods (TSA-Net [24], HDNet [26], MST [25], CST [27]) and five DUN methods (DGSMP [39], HerosNet [42], DAUHST [44], PADUT [45], RDLUF [46]). We present the PSNR and SSIM results for all comparison methods, with the best and second-best reconstruction results for all test scenes highlighted in red and blue, respectively. In addition, we calculate the number of parameters (Params) and floating-point operations (FLOPs) to evaluate the model complexity of all comparison methods. As seen in Table 2, our method achieves the best average test results and optimal performance across all scenarios. Specifically, our method reached PSNR and SSIM metrics of 42.98 dB and 0.985, respectively. Compared to the next best method, our approach shows an average improvement of 0.2dB in PSNR and 0.001 in SSIM. These objective results validate the effectiveness of the proposed method on the ARAD_1K dataset.

**Figure 10:**
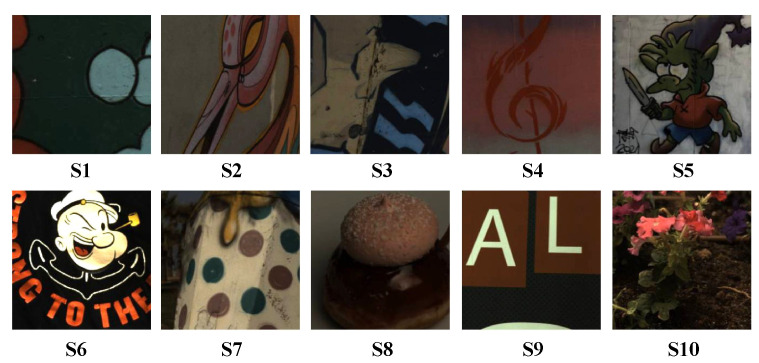
RGB images of ten test scenes from the ARAD_1K dataset.

**Figure 11:**
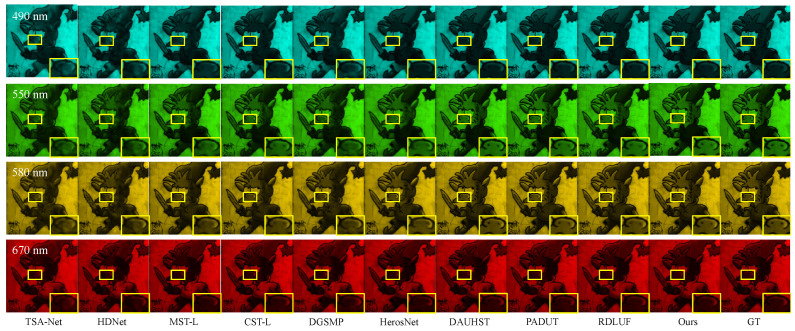
Comparison of the reconstruction results for scene S5 in the ARAD_1K test dataset using different methods. The enlarged regions help to compare the reconstruction results better.

To illustrate the qualitative results on the ARAD_1K test dataset, we present a subjective comparison of the reconstruction outcomes for the test scenes using different methods. The RGB images of all test scenes from the ARAD_1K dataset are shown in Figure 10. The subjective comparison of the reconstruction results for scene S5 is shown in Figure 11. We select reconstruction results from four different spectral bands to compare the subjective performance of all methods. To facilitate the comparison of differences in reconstruction results, we crop and enlarge the yellow box areas in each spectral band image. As shown in Figure 11, our method produces reconstruction results with fewer artifacts and clearer structural textures compared to other methods. This further validates the effectiveness of our approach on the ARAD_1K test dataset.

Additionally, to further compare the spectral consistency of reconstruction results from different methods, we also select a small region from test scene S5 for evaluation, as shown in Figure 9b. We present the spectral density curves of different reconstruction results and calculate the correlation coefficients between the reconstructed results and the ground truth data. Our method’s reconstruction results are the closest to the ground truth spectral density curve and achieve the highest correlation coefficient, further validating the accuracy of our reconstruction.

### 4.4. Real Data Results

**Figure 12:**
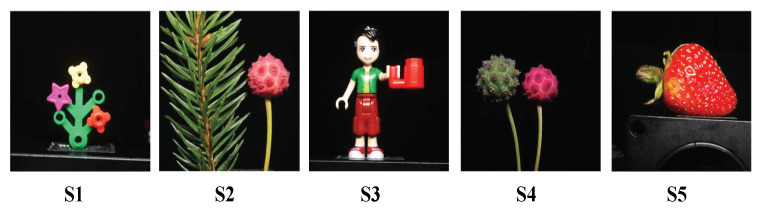
RGB images of five real-world test scenes.

**Figure 13:**
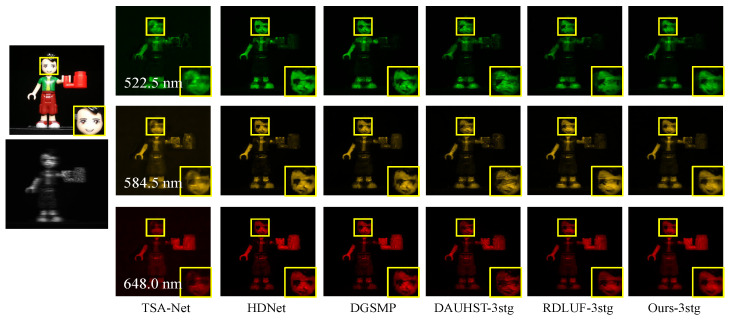
Subjective comparison of reconstruction results for three different spectral bands in real scene S3 using different methods. Enlarged areas are used to compare the differences in reconstruction results more clearly.

To validate the effectiveness of our method in real-world scenarios, we conduct tests on a real-world scene dataset. We compare our method with five advanced methods, including two E2E methods (TSA-Net [24], HDNet [26]) and three DUN methods (DGSMP [39], DAUHST [44], RDLUF [46]). Due to the large size of the test scenes, our method and other DUN methods are all compared using the same three-stage network. The RGB images of the five test scenes from the real-world scene dataset are shown in Figure 12. Since the dataset lacks ground truth data, we compare the reconstruction results of different methods by referencing the RGB images. We select scene S3 to present the subjective reconstruction results. As shown in Figure 13, our method produces smoother and clearer appearances in three different spectral bands. Notably, for the yellow-marked facial area, our reconstruction results retain structural content with fewer reconstruction artifacts. This demonstrates the effectiveness of our proposed method for real-world test data.

### 4.5. Ablation Study

To validate the impact of the number of stages on the overall performance of our algorithm, we conduct ablation experiments with different stage counts. As shown in Table 3, we evaluate the model complexity and average reconstruction performance of our algorithm with varying stage numbers on the CAVE and KAIST simulation datasets. Since our algorithm maintains parameter sharing within the network structure of each stage, the number of model parameters does not change with the number of stages. However, the number of floating-point operations (FLOPs) increases with the number of stages. The results indicate that the performance of our method improves with the increase in the number of stages. To balance model effectiveness and complexity, we ultimately adopt nine stages for the overall model configuration.

To verify the effectiveness of different modules in our method, we conduct ablation experiments with models composed of different structures. All experiments utilize models with nine stages and are tested on the CAVE and KAIST simulation datasets. Table 4 shows the average test reconstruction results and model complexity for different model configurations. We use the basic spatial–spectral Transformer structure as a baseline for comparison, as shown in row (a) of Table 4. To evaluate the effectiveness of different modules in enhancing reconstruction, we conduct four experiments: using only the SAET module, combining the SAET and WIntraF modules, combining the SAET and WInterF modules, and combining all modules. The results of these experiments are presented in rows (b) to (e) of Table 4, respectively. Specifically, from the results of row (b) in Table 4, it is evident that compared to the baseline model, the SAET model significantly enhances reconstruction performance, with improvements of 0.25 dB in PSNR and 0.001 dB in SSIM. This demonstrates the improvement in the overall model’s expressive capability brought by our proposed sparse spatial–spectral Transformer network structure. Additionally, comparing rows (c) and (d) with row (b) in Table 4 shows that the model combining SAET and WIntraF achieves a 0.11 dB improvement in PSNR over the SAET-only model, while the model combining SAET and WInterF achieves a 0.14 dB improvement in PSNR over the SAET-only model. These results clearly validate the effectiveness of the wavelet-based intra-stage and inter-stage fusion modules. Finally, the reconstruction results of the complete model in row (e) verify the performance enhancement of the proposed model across various component structures.

To validate the impact of different sparsity coefficients in the sparse spatial–spectral Transformer structure, we conduct ablation experiments with various sparsity coefficients. As shown in Table 5, the spatial Top-k and spectral Top-k rows represent the spatial and spectral sparsity coefficients, respectively. Different sparsity coefficients indicate the proportion of sparse elements in the correlation matrix relative to the total elements. When the spatial and spectral sparsity coefficients are both set to 1, all elements are selected as sparse elements, making the model structure identical to the original spatial–spectral Transformer structure. According to Table 5, the optimal reconstruction performance is achieved when the spatial sparsity coefficient is 1/3 and the spectral sparsity coefficient is 4/5, resulting in a 0.25 dB improvement in PSNR compared to the original spatial–spectral Transformer structure. Therefore, in the overall algorithm, we select 1/2 and 4/5 as the sparsity coefficients for the spatial and spectral dimensions, respectively.

## 5. Conclusions

In this study, we propose a deep unfolding network that hybridizes sparse Transformer and wavelet fusion for the snapshot compressive imaging of hyperspectral images (HSIs). Firstly, since not all elements in hyperspectral images are correlated in the spatial and spectral dimensions, we introduce a spatial–spectral sparse Transformer technique to enhance the feature representation capability of the algorithm. Then, to address the issue of feature information loss due to scale transformation within stages, we propose a wavelet-based intra-stage feature fusion method. Finally, we introduce a wavelet-based inter-stage feature fusion method to tackle feature information loss caused by signal-to-feature conversions between stages. Experiments on various simulated and real datasets further validate that the proposed algorithm achieves superior hyperspectral image reconstruction results. However, the current method’s utilization of wavelet transforms is still preliminary. In future work, we will explore how to learn the sparsity coefficients to better accommodate different model structures. Additionally, we will continue to explore how to utilize wavelet transform features better to design network structures, thereby further improving the algorithm’s performance.

## Figures and Tables

**Table 1:** Comparison of results using the KAIST test scenes, with the PSNR metric (in dB) presented as the upper entry and the SSIM metric as the lower entry in each cell. The best results are highlighted in red, and the second-best results are highlighted in blue.

Algorithms	Params	FLOPs	S1	S2	S3	S4	S5	S6	S7	S8	S9	S10	Average
TSA-Net [24]	44.25 M	110.06 G	32.03	31.00	32.25	39.19	29.39	31.44	30.32	29.35	30.01	29.59	31.46
			0.892	0.858	0.915	0.953	0.884	0.908	0.878	0.888	0.890	0.874	0.894
HDNet [26]	2.37 M	154.76 G	35.14	35.67	36.03	42.30	32.69	34.46	33.67	32.48	34.89	32.38	34.97
			0.935	0.940	0.943	0.969	0.946	0.952	0.926	0.941	0.942	0.937	0.943
MST-L [25]	2.03 M	28.15 G	35.40	35.87	36.51	42.27	32.77	34.80	33.66	32.67	35.39	32.50	35.18
			0.941	0.944	0.953	0.973	0.947	0.955	0.925	0.948	0.949	0.941	0.948
CST-L [27]	3.00 M	27.81 G	35.82	36.54	37.39	42.28	33.40	35.52	34.44	33.83	35.92	33.36	35.85
			0.947	0.952	0.959	0.972	0.953	0.962	0.937	0.959	0.951	0.948	0.954
DGSMP [39]	3.76 M	646.65 G	33.26	32.09	33.06	40.54	28.86	33.08	30.74	31.55	31.66	31.44	32.63
			0.915	0.898	0.925	0.964	0.882	0.937	0.886	0.923	0.911	0.925	0.917
HerosNet [42]	11.75 M	446.29 G	35.75	35.40	34.07	38.59	33.31	35.58	33.27	33.75	34.04	33.18	34.69
			0.972	0.968	0.966	0.987	0.969	0.977	0.963	0.971	0.967	0.968	0.971
DAUHST [44]	6.15 M	79.50 G	37.25	39.02	41.05	46.15	35.80	37.08	37.57	35.10	40.02	34.59	38.36
			0.958	0.967	0.971	0.983	0.969	0.970	0.963	0.966	0.970	0.956	0.967
PADUT [45]	5.38 M	90.46 G	37.36	40.43	42.38	46.62	36.26	37.27	37.83	35.33	40.86	34.55	38.89
			0.962	0.978	0.979	0.990	0.974	0.974	0.966	0.974	0.978	0.963	0.974
RDLUF [46]	1.89 M	231.09 G	37.74	40.76	43.05	47.59	36.93	37.54	38.34	35.57	42.18	34.77	39.45
			0.967	0.979	0.981	0.992	0.978	0.978	0.971	0.974	0.982	0.964	0.977
Ours	2.25 M	121.43 G	37.85	40.80	43.10	48.12	37.48	37.52	38.63	36.41	42.04	35.62	39.76
			0.970	0.980	0.982	0.993	0.980	0.979	0.971	0.979	0.982	0.970	0.979

**Table 2:** Comparison of results using the ARAD_1K test scenes, with the PSNR metric (in dB) presented as the upper entry and the SSIM metric as the lower entry in each cell. The best results are highlighted in red, and the second-best results are highlighted in blue.

Algorithms	Params	FLOPs	S1	S2	S3	S4	S5	S6	S7	S8	S9	S10	Average
TSA-Net [24]	44.25 M	110.06 G	33.79	28.38	27.11	33.36	25.85	25.09	29.49	20.88	21.34	31.88	27.72
			0.950	0.877	0.886	0.924	0.831	0.671	0.888	0.687	0.782	0.875	0.837
HDNet [26]	2.37 M	154.76 G	35.18	28.96	28.54	35.54	26.58	31.40	30.22	29.99	29.77	32.93	30.91
			0.935	0.862	0.875	0.918	0.816	0.823	0.874	0.793	0.884	0.885	0.866
MST-L [25]	2.03 M	28.15 G	37.89	31.44	31.06	36.81	29.79	35.05	32.83	32.62	34.09	34.99	33.66
			0.962	0.903	0.917	0.935	0.897	0.906	0.921	0.858	0.941	0.923	0.916
CST-L [27]	3.00 M	27.81 G	41.06	34.09	33.40	39.25	32.18	38.78	35.16	34.62	37.06	36.72	36.23
			0.978	0.938	0.948	0.957	0.935	0.955	0.953	0.901	0.972	0.947	0.948
DGSMP [39]	3.76M	646.65G	37.10	29.97	29.44	36.30	28.20	34.80	31.18	31.51	32.05	33.92	32.45
			0.959	0.886	0.908	0.935	0.875	0.901	0.908	0.840	0.928	0.912	0.905
HerosNet [42]	11.75 M	446.29 G	38.17	33.27	32.11	39.31	29.44	33.90	32.72	32.22	33.18	33.96	33.83
			0.981	0.958	0.955	0.982	0.929	0.951	0.948	0.926	0.977	0.947	0.955
DAUHST [44]	6.15 M	79.50 G	45.94	40.53	39.26	46.85	38.26	43.67	40.44	38.94	44.41	40.62	41.89
			0.991	0.978	0.978	0.990	0.979	0.988	0.980	0.959	0.990	0.974	0.980
PADUT [45]	5.38 M	90.46 G	46.65	41.20	39.85	47.79	38.93	44.38	41.07	39.53	45.45	41.05	42.59
			0.992	0.981	0.981	0.992	0.981	0.990	0.982	0.963	0.993	0.977	0.983
RDLUF [46]	1.89 M	231.09 G	46.50	41.15	40.35	48.16	38.77	44.61	41.09	40.21	45.98	41.01	42.78
			0.992	0.980	0.982	0.993	0.981	0.991	0.982	0.970	0.993	0.976	0.984
Ours	2.25 M	121.43 G	46.72	41.47	40.41	48.18	39.12	44.73	41.16	40.53	46.29	41.17	42.98
			0.993	0.982	0.982	0.993	0.983	0.992	0.983	0.970	0.995	0.977	0.985

**Table 3:** The computational complexity and average reconstruction performance across various numbers of network stages on the KAIST test scenes.

Stage Number	Params (M)	FLOPs (G)	PSNR (dB)	SSIM
1	2.25	26.10	37.05	0.964
3	2.25	39.72	38.11	0.971
5	2.25	66.96	38.74	0.974
7	2.25	94.20	39.48	0.977
9	2.25	121.43	39.76	0.979

**Table 4:** The effectiveness of different components.

Setting	SAET	WIntraF	WInterF	Params (M)	FLOPs (G)	PSNR (dB)	SSIM
(a) (Base)				1.85	109.51	39.33	0.977
(b)	*√*			1.85	109.51	39.58	0.978
(c)	*√*	*√*		1.90	112.34	39.69	0.978
(d)	*√*		*√*	2.21	118.60	39.72	0.978
(e) (Ours)	*√*	*√*	*√*	2.25	121.43	39.76	0.979

**Table 5:** The impact of spatial and spectral sparsity coefficients in the SAET module, where Top-k represents the proportion of selected elements to all elements.

**Spatial Top-k**	1	1/2	1/2	1/2	1/3	1/4	2/3
**Spectral Top-k**	1	1	4/5	3/4	4/5	4/5	4/5
**PSNR (dB)**	39.33	39.53	39.52	39.45	39.58	39.32	39.45
**SSIM**	0.977	0.975	0.978	0.977	0.978	0.976	0.977

## Data Availability

Data are contained within the article.
